# ASSESSING THE IMPACT OF A GAME-CENTERED MOBILE APP ON COMMUNITY-DWELLING OLDER ADULTS’ HEALTH ACTIVATION

**DOI:** 10.1093/geroni/igz038.1229

**Published:** 2019-11-08

**Authors:** Jason Crandall, Matthew Shake, Uta Ziegler

**Affiliations:** 1 Western Kentucky University Center for Applied Science in Health and Aging, Bowling Green, Kentucky, United States; 2 Western Kentucky University Department of Psychological Sciences, Bowling Green, Kentucky, United States; 3 Western Kentucky University School of Engineering and Applied Science, Bowling Green, Kentucky, United States

## Abstract

The objective of this investigation was to evaluate the impact of a game-centered mobile app (Bingocize®) on older adults’ knowledge, skill, and confidence for managing aspects of their healthcare. Rural community-dwelling older adults (N=85) with mobility, not engaged in any structured exercise program, used the app in a group setting for 10 weeks, twice per week, for one hour. Participants were randomly assigned to (a) a version that included health education, or (b) health education and an exercise component. The Patient Activation Measure (PAM-10) was used to assess group changes in knowledge, skill, and confidence for managing aspects of their healthcare. Two (Group: Exercise + Health Education vs. Health Education-only) x Two (Time: Pre- vs. Post-intervention) analyses of variance, with significance p<.05, was used for statistical analysis. Results: PAM-10 values significantly increased from pre- to post-intervention for both groups, as did knowledge of the health topics (all p < 0.05). Attendance was >93% in both groups. Bingocize® engendered high attendance and improved health activation of older adults; however, additional research is needed to examine whether changes in activation result in long-term changes in health behavior. The Bingocize® mobile app is an enjoyable and effective way to increase health activation in community-dwelling older adults.

